# Therapeutic Applications of Herbal Medicines for Cancer Patients

**DOI:** 10.1155/2013/302426

**Published:** 2013-07-11

**Authors:** Shu-Yi Yin, Wen-Chi Wei, Feng-Yin Jian, Ning-Sun Yang

**Affiliations:** Agricultural Biotechnology Research Center, Academia Sinica, No. 128, Section 2, Academia Road, Nankang, Taipei 115, Taiwan

## Abstract

Medicinal herbs and their derivative phytocompounds are being increasingly recognized as useful complementary treatments for cancer. A large volume of clinical studies have reported the beneficial effects of herbal medicines on the survival, immune modulation, and quality of life (QOL) of cancer patients, when these herbal medicines are used in combination with conventional therapeutics. Here, we briefly review some examples of clinical studies that investigated the use of herbal medicines for various cancers and the development of randomized controlled trials (RCTs) in this emerging research area. In addition, we also report recent studies on the biochemical and cellular mechanisms of herbal medicines in specific tumor microenvironments and the potential application of specific phytochemicals in cell-based cancer vaccine systems. This review should provide useful technological support for evidence-based application of herbal medicines in cancer therapy.

## 1. Clinical Uses of Herbal Medicine with Anticancer Effects

A range of clinical studies have indicated that a spectrum of anticancer activities from various herbal medicines can be detected. In this section, we have organized and classified the clinical use of a number of herbal medicines according to their suppressive effect on specific cancer types (Table 1). 

### 1.1. For Breast Cancer

Although a specific role for vitamins and selenium in the prevention of breast cancer has not been established, some anticancer activities have been shown* in vitro* [1–3]. In a randomized controlled trial, 2972 patients with invasive or noninvasive breast carcinoma received either 200 mg of vitamin A preparation (Fenretinide) per day or no therapy. At 97 months posttreatment there was a significant reduction in recurrence of local breast cancer in premenopausal women (HR: 0.65; 95% CI: 0.46–0.92). However, no significant difference in metastasis or overall survival time could be demonstrated [4]. Interestingly, other studies have shown that long-term uptake of vitamin E may in fact have a negative effect on breast cancer patients [5, 6]. Currently, their rule seems to cause malabsorption or maldigestion in cancer patients suffering from a concomitant illness, moreover, providing patients adopt a balanced and healthy diet [4, 7].

Phytoestrogens are classified into water-soluble isoflavones and lipophilic lignans. Isoflavones are found in high abundance in soy beans, and lignans are found in linseeds wheat, fruit, flaxseeds, and vegetables [8, 9]. Among six related clinical trials conducted so far, only one concluded that isoflavone  was  associated with a reduced risk of breast cancer [10]. Soy-derived phytoestrogens are popularly recommended for treating postmenopausal symptoms in women with breast cancer undergoing tamoxifen therapy. The principal constituents of soy bean plant extracts, including isoflavones genistein and daidzein, are structurally similar to 17*β*-estradiol and can confer weak estrogenic effects [11]. However, there is no evidence to support the recommendation of use of phytoestrogens either in treating breast cancer or for easing climacteric symptoms [12].

Investigations of traditional Chinese medicines (TCM) have uncovered a number of antibreast cancer agents, although most of their mechanisms of action have not yet been elucidated. These TCM herbs with antibreast cancer activities can be classified into six categories: alkaloids [13, 14], coumarins [15, 16], flavonoids and polyphenols [17, 18], terpenoids [19], quinone [20], and artesunate [21] (Table 1). Some of these phytochemicals, such as curcumin and artemisinin, have well-known chemical structures. Compounds in these categories have been taken as health foods or dietary supplement for decades. However, evidence-based *in vivo* studies and clinical trials are still recommended for routine public use or specific clinical applications. 

### 1.2. For Prostate Cancer

Prostate cancer is characterized by a long latency period, a strong dietary influence, and limited treatment strategies for the advanced disease; therefore, many patients turn to complementary and alternative medicine (CAM) with the belief that these medicines represent a viable therapeutic option that may be free of adverse side effects [22]. This folkloric belief, strongly upheld in many Asian cultures, needs to be substantiated with systematic, evidence-based research. Vitamins, including vitamins A–D and retinoids, are organic compounds that cannot be synthesized by humans and must be ingested to maintain homeostasis and prevent various metabolic disorders [23]. Emerging evidence indicates that inflammation might have a crucial role in the genesis of prostate carcinoma [24–26]. A number of clinical trials have sought to evaluate the anti-inflammatory activities of vitamins on prostate carcinogenesis [27]. Despite a lack of convincing evidence, vitamin and mineral supplements are used extensively by patients that are diagnosed with prostate cancers [28, 29]. The belief is that such supplements might actually prevent or treat inflammation-associated disease and at the very least not cause harm [22]. Among smokers, daily ingestion of >100 IU of vitamin E was reported to produce a 56% reduction in risk in lethal or advanced prostate cancer relative to nonusers [30]. On the other hand, a selenium and vitamin E chemoprevention trial (SELECT) that aimed to determine whether vitamin E and/or selenium supplementation could reduce prostate carcinogenesis showed that dietary supplementation with vitamin E in fact statistically increased the risk of prostate cancer among healthy men [31, 32]. Together these clinical data suggest that the application of vitamin E may be specific for only treating inflammation-associated features in prostate cancer patients, rather than affecting anticancer activity. Future studies are needed to address such an apparent contradiction between effects in healthy and cancer populations.

The use of medicinal herbs and their derivative herbal extracts that contain numerous polyphenolic compounds may contribute to the lower incidence of prostate cancer in Asian populations relative to Caucasians and African Americans [33]. Many polyphenols (e.g., isoflavones) are phytoestrogens that can bind to estrogen receptors and elicit estrogenic effects in target tissues or organs. Some specific compounds, in particular the four active polyphenolic compounds in green tea, epicatechin, epigallocatechin, epicatechin-3-gallate, and epigallocatechin-3-gallate (EGCG), the soy isoflavones, as well as *Scutellaria baicalensis*, *β*-carotene, and the lycopenes have all been studied for their effect on prostate carcinoma [22]. Using specific bioassays, EGCG in green tea was demonstrated to arrest LNCaP and DU145 prostate cancer cells at the G0-G1 phase of the cell cycle [34] and inhibit metalloproteinase *in vitro*, although the effect was achieved at a much higher concentration than the serum levels detected in humans who consumed moderate amounts of green tea [35]. In a Phase II study of green tea in the treatment of patients with androgen-independent metastatic prostate carcinoma, one patient achieved a prostate-specific antigen (PSA) response of  >50% that lasted for approximately one month. Patients, however, suffered marked symptoms of toxicity in this study, most notably diarrhea, nausea, and fatigue. Investigators concluded that green tea has limited antineoplastic activity, as defined by a decline in PSA levels, against androgen-independent prostate cancers [36]. For soy isoflavones, they have been shown to inhibit 5*α*-reductase activity, the enzyme that is functionally responsible for the conversion of testosterone to the more potent androgen dihydrotestosterone [37]. Several pre-clinical randomized studies have also evaluated the potential therapeutic effects of soy isoflavones as chemopreventive agents [22]. For *Scutellaria baicalensis*, it contains very high levels of *baicalin*, a flavone glycoside that inhibits enzymatic synthesis of eicosanoids, which are important mediators of inflammation and prostate tumor cell proliferation [38]. Baicalein (a flavone) impairs the proliferation of androgen-independent PC-3 and DU145 prostate cancer cells in culture and induces cell-cycle arrest at the G0-G1 phase [39] and apoptosis at concentrations achievable in humans [40]. Baicalein drastically suppressed the expression of specific androgen receptor in prostate cancer at clinically achievable concentrations [40]. For lycopenes, although most studies have used mixtures of tomato products, the association of decreased prostate cancer risk and tomato product intake has led to the specific focus on the use and effect of lycopene [41]. Chen and colleagues studied the effect of lycopene levels and measures of oxidative damage in patients undergoing prostatectomy for localized prostate cancer [42]. They found that oxidative damage in lymphocytes from these patients was diminished after dietary intervention compared with pretreatment levels and that the prostate tissues in treated patients revealed significantly decreased levels of PSA and less oxidative damage. Since it still remains uncertain whether lycopene itself causes the effect, or whether a more complex food extract is responsible, additional randomized trials are needed to assess the efficacy of lycopene in chemoprevention activities.

Sales of the herbal extract PC-SPES as a dietary supplement for “prostate health” began in the mid-1990s. The name PC-SPES is derived from PC for “prostate cancer” and “spes,” the Latin word for hope. This botanical mixture is used primarily for treatment of prostate carcinoma [43]. The formulation contains extracts of eight herbs, *Ganoderma lucidum*, *Scutellaria baicalensis*, *Rabdosia rubescens*, *Isatis indigotica*, *Dendranthema morifolium*, *Serenoa repens*, *Panax notoginseng*, and *Glycyrrhiza uralensis*, that were selected based on either their use in Chinese medicinal therapy for urinary problems or their antitumor efficacy against cancer cell lines [44]. In addition, a series of clinical studies have described the effects and mechanisms of PC-SPES activity [45]. Although the therapeutic application of PC-SPES seemed to be promising, unfortunately, PC-SPES was recalled and withdrawn from the market because certain batches of testing PC-SPES samples were found to be contaminated with US Food and Drug Administration-(FDA-)controlled prescription drugs. To our knowledge, the FDA has not approved so far the use of PC-SPES in cancer treatment. More evidence and correlative information to demonstrate the *in vitro *and clinical efficacy of this herbal mixture are needed.

Lin et al. [46] showed that *Wedelia chinensis* (Asteraceae), an oriental medicinal herb containing various compounds such as indole-3-carboxylaldehyde, wedelolactone, luteolin, and apigenin, is capable of suppressing androgen activity. Moreover, oral administration of *W. chinensis* extract impeded prostate cancer tumorigenesis. This anticancer action of *W. chinensis* extract was subsequently demonstrated to be due to three active compounds that can inhibit the androgen receptor (AR) signaling pathway [47]. Recently, our own study showed that a different set of phytochemicals extracted from *W. chinensis* plants can confer potent and specific anti-inflammatory bioactivities *in vitro* and *in vivo*. These activities resulted in strong anticolitis activities in test mice [48]. Future studies of *W. chinensis* for chemoprevention or as a complementary medicine against prostate cancer in humans are warranted.

### 1.3. For Lung Cancer

Lung cancer is one of the most deadly cancers, and the lung is a common site of metastasis of tumors from other tissues in the body. Standard chemotherapy regimes often have limited survival benefits due to the severe toxicity [49, 50] of the various anticancer agents, such as gemcitabine, paclitaxel, docetaxel, etoposide, and vinorelbine. Recent reports have suggested that herbal medicines and their phytochemicals which seem to have lower or little toxicity may provide an attractive strategy for lung cancer therapy. Traditionally, herbal plants such as *Platycodon grandiflorum* (Campanulaceae), *Morus alba* (Moraceae), *Prunus armeniaca* (Rosaceae), *Rhus verniciflua* (Anacardiaceae), *Perilla frutescens* (Labiatae), *Stemona japonica* (Stemonaceae), *Tussilago farfara* (Compositae), and *Draba nemorosa* (Brassicaceae) have been used to treat lung cancer [51]. Clinically, the proportion of patients that use herbal medicines as adjuvants alongside conventional (e.g., chemotherapy) treatment for lung cancer is as high as 77% [52]. Herbs are mainly used in lung cancer to reduce therapy-associated toxicity and cancer-related symptoms and sometimes to directly increase anticancer effects [4]. However, it is important to note that some CAM methods or remedies may have adverse effects or reduce the efficacy of conventional treatment, and the primary justification for use of traditional herbal medicines remains empirical evidence, case studies, and hypothetical physiological effects [4]. 

### 1.4. For Liver Fibrosis and Cancer

Liver fibrogenesis is a gradual process of increased secretion and decreased degradation of extracellular materials, which can be initiated by activation of hepatic stellate cells (HSCs) [64, 65]. The number of deaths due to hepatocellular carcinoma (HCC) has steadily increased over the last decade. Unfortunately, there are no successful, clinically satisfactory therapies for patients suffering HCC. Herbal medicines are being considered as one possible strategy against liver fibrosis and HCC. Three medicinal herbs are already used as official drugs in China, Japan, and other parts of Asia. Different chemically induced fibrosis models were designed using the rat liver system to assess the preventive effects of specific herbal extracts on liver fibrosis. Formulations assessed include Inchin-ko-to (TJ-135) [53], Yi Guan Jian (YGJ) [53], Yi Guan Jian (YGJ) [54], Fufang-Liu-Yue-Qing [55], and Danggui Buxue Tang (DBT) [56]. In 2007, Luk et al. [57] provided a systematic review of the mechanisms of action of medicinal herbal compounds, such as salvianolic acid B (SAB), oxymatrine, and curcumin in the treatment of hepatic fibrogenesis and carcinogenesis. Although some of these herbal medicines, such as YGJ, are traditionally used to treat human liver fibrosis, the therapeutic or clinical anticancer effect of these herbal mixtures in liver tissue has not been fully elucidated. The further identification of as yet unknown effective components in these herbal extract is critical for their pharmacological use and improvement. 

A combination of 10 herbs (named compound 861), including *Salvia miltiorrhiza* (sage), *Astragalus membranaceus*, and *Spatholobus suberectus* known in TCM as the “king herb” components of the formula, that is, the herbs that are pharmacologically active, and seven others (modifiers of toxicity that act synergistically with the king herbs to improve immune function), has been tested in a number of experimental studies for antifibrotic properties. Two uncontrolled open trials of 60 and 22 patients with chronic hepatitis B who were treated with compound 861 reported a beneficial effects on liver fibrosis, with the majority of treated patients showing both clinical and histological improvement [58, 59]. Since these clinical studies of compound 861 did not satisfy quality control criteria, clinicians consider that additional well-designed trials are needed for routine and authorized clinical use of compound 861 for the treatment of hepatitis B-induced liver fibrosis.

A large number of clinical reports have indicated the therapeutic effects of one Japanese traditional medicine (*kampo yaku*) Sho saiko-to (TJ-9). This medicine is a combination of seven herbs traditionally used for treating liver diseases [66, 67]. However, little clinical data on the efficacy of TJ-9 in preventing human liver cancer has been reported. In a long-term (5 years) randomized controlled study, patients that were positive for hepatitis B surface antigen (HBsAg) received a dose of 7.5 g/day aqueous TJ-9 extract together with the standard treatment using interferon. Upon followup, the cumulative development of hepatocellular carcinoma (HCC) was found to be significantly lower than that of the controls (i.e., patients without TJ-9 treatment) [60]. Unfortunately, TJ-9 is contraindicated for patients with hepatic cirrhosis or acute respiratory failure in Japan, because some of these patients were found to contract interstitial pneumonia after drug administration [68]. Therefore, well-designed future trials that can address the specificity of TJ-9 or its major active components in inhibition or suppression of the progression of viral hepatitis-induced hepatocellular carcinoma or metabolic liver cancers are needed [69].

### 1.5. For Pancreatic Cancer

Smoothened (SMO), a component of the sonic hedgehog homology (SHH) signaling pathway, has been shown to play a key role in the cellular behavior of cancer stem cells [70]. The deregulation of SHH was considered as an important factor that can drive or maintain the progression of pancreatic cancer [71]. There are some SMO antagonists that, such as GDC-0449, IPI-926, XL-139, and PF-04449913, are being evaluated with high hope for treatment of pancreatic cancers [61]. Cyclopamine, a steroidal alkaloid extracted from *Veratrum californicum*, can efficiently inhibit SHH signaling by directly binding to the 7-helix bundle of the SMO protein. This complex can further impact upon the function of 12-transmembrane receptor patched-1 (PTCH-1) and thereby influence the structure of SMO [62]. It needs to be noted here that cyclopamine not only can weaken the recruitment of bone marrow precursor cells (BMPCs) into cancer cells, but also can reduce the formation of tumor vasculature [63]. The cancerous vascular system becomes unstable after treatment with cyclopamine due to the expression of angiopoietin-1, an angiogenic factor found in the tumor microenvironment, which is under the regulation of SHH. Cyclopamine has been explored as an SMO activity suppression agent and to arrest the growth of pancreatic tumors [63]. Encouraging findings suggest that this phytochemical obtained from a traditionally used TCM herb should be systematically explored in the future for efficacious SMO-targeting anticancer drugs.

## 2. Use of Herbal Supplements as Adjuvants in Conventional AntiCancer Therapies

**Figure 1:**
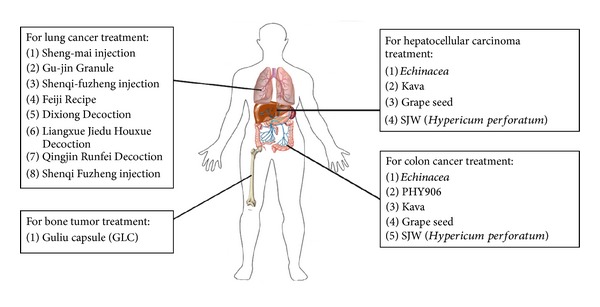
Medicinal herb “extracts” or “formulations” being tested as adjuvant treatments for chemo- or radiotherapies against various cancers.

Numerous Chinese herbal medicines are being used in combination with chemotherapy or radiotherapy to improve the efficacy of cancer therapy and reduce side effects and complications (Figure 1), although this practice is highly frowned upon by many western physicians. Understanding of the use of specific herbal medicines as adjuvants to conventional therapy, therefore, needs to be increased in consultation and coordination with physicians and other health care providers. This section outlines evidence for use of herbal medicines as adjuvants to conventional drug-based, chemo- or radiotherapy regimes in cancer treatment. On the other hand, this section also summarizes the challenges or limitations for clinical use of these herbal medicines.

### 2.1. Common Use of Herbal Medicines as Adjuvant Treatment in Chemo- or Radio-Cancer Therapy

For the above adjuvant anticancer therapy studies, herbal medication in general was applied as a combination therapy with the conventional chemotherapy to hopefully increase the therapeutic benefit and quality of life (QoL) as well as to decrease the side effects or complications. Between 28% and 98% of ethnic Chinese cancer patients in Asia [72–74] and 25% to 47% of those living in North America are reported to have used herbal medicines as part of their cancer care [75, 76]. Although a number of herbal medicines have been found to be adjunctive in chemo- and radiotherapy, most clinical trials or studies have been reported mainly, if not only, in China or other Asian countries and they are virtually not cited on PubMed. In 2010, Qi and colleagues [77] provided a systematic review of Chinese herbal medicines in clinical trials, mainly as adjuvant treatments to reduce complications and side effects of chemo- or radiotherapy. Several traditionally used Chinese herbal medicines, including astragalus [78, 79], Turmeric (curcumin) [80–82], Ginseng [83–85], TJ-41 (Bu-Zhong-Yi-Qi-Tang) [86, 87], PHY906 [74, 88–90], Huachansu [91, 92], and Kanglaite [93, 94], are commonly used by cancer patients to either “treat” cancer and/or “reduce the toxicity” induced by chemotherapy or radiotherapy. Preclinical and clinical studies have indicated that these herbal medicines may possess a number of advantages in terms of suppression of tumor progression, by increasing the sensitivity of chemo- and radiotherapeutics, improving immune system function, and easing the tissue/physiology damage caused by chemo- and radiotherapeutics. However, most studies to date are empirical (i.e., not well controlled) clinical studies or observations that mainly report reduction in side effects and complications during or after chemotherapy and radiotherapy. Some traditional herbal formulations, including Bojungikki-tang [87], Kang-Fu-Zhi-Tong [95], PHY906 [88], Xiao-Chai-Hu-Tang, Huang-Lian-Jie-Du-Tang, and Yin-Chen-Wu-Ling-San [96], have been observed, “detected,” or “claimed” to protect liver function, reduce cancer-related fatigue and pain, improve respiratory tract infections and gastrointestinal side effects, and even ameliorate the symptoms of cachexia. Often, these clinical results do not meet the standard US FDA requirements for clinical trials, but they may still offer some insight into traditionally used herbal medicines as adjuvant treatments for cancers. They may also provide useful pointers for the development of future botanical drugs as cancer primary or adjuvant therapies [74, 77, 97]. 

### 2.2. For Lung Cancer

In a randomized controlled trial (RCT) with 63 patients with non-small-cell lung cancer (NSCLC), Sheng-mai Injection (Ya'an Sanjiu Pharmaceutical Co., China) and Gu-jin Granule (Jiangyin Tianjiang Pharmaceutical Co., China) were observed to enhance median survival time (*P* = 0.014) and response rate increase to 48.5% (16/33), compared to the untreated control (32.2% = 9/28) in the control group (*P* = 0.0373), while all test groups were treated with a combination of navelbine and cisplatin (NP) chemotherapy [98]. In another clinical trial with Shenqi-fuzheng injection (Lizhu Co., China) among 232 NSCLC patients enrolled, herbal injection significantly improved the response rate and QoL of lung cancer patients, evaluated by using the QoL scale of European Organization for Research on Treatment of Cancer (QLQ-C30) [99]. Furthermore, the randomized controlled trial for Feiji Recipe treatment was also observed to enhance the clinical therapeutic efficacy and alleviate side effects of chemotherapy [100], as shown with an increase on scores in role, social, and economic status (*P* < 0.05 or *P* < 0.01), again based on QLQ-C30 questionnaire [101]. Recently, Xu et al. [102] applied a high quality of clinical trial methodology to examine the effect of TCM on improving QoL of postoperative non-small-cell lung cancer (NSCLC) patients. They clearly presented the design and protocol for a placebo-controlled, double-blinded RCT and were able to systematically provide evidence for the effectiveness of chemotherapy combined with TCM in improving QOL of postoperative NSCLC patients. The result is expected to provide support for integrative optimization of “combined” treatment of lung cancer patients. 

One of the major risks of conventional treatment in lung cancer patients is radiation pneumonitis, caused by radiotherapeutic intervention [103]. Increasing evidence has been reported on the beneficial efficacy of certain herbal medicines such as Dixiong Decoction [104], Liangxue Jiedu Huoxue Decoction [105], Qingjin Runfei Decoction [106], and Shenqi Fuzheng injection [107] (Figure 1). These herbal formulations were reported to significantly lower the incidence of radiation pneumonitis and improve clinical radiographic physiologic (CRP) dyspnea score and the Radiation Therapy Oncology Group (RTOG) grading score, in groups of NSCLC patients undergoing radiotherapy treatment. These findings also revealed some of the possible adverse effects and potential uses of specific herbal medicines in combinational therapy alongside conventional chemotherapy. The broad range and heterogeneity of herbal medicine intervention and the resultant effects still pose a challenge to high-powered analysis of specific herbal medicines and their applications for evidence-based use in cancer therapies. Therefore, high level quality control to ensure consistent batch preparation and systematic pharmacokinetic studies are required for all test herbal medicines and their activity against lung cancer [108], not only in human studies, but also in experimental animal systems to support evidence-based application.

### 2.3. For Colon Cancer

In oncology, drug interactions are important because of the narrow therapeutic index and inherent toxicity of many anticancer agents [109]. Previous studies indicated that the activity of cytochrome P450 enzymes (CYP enzymes) in the gastrointestinal wall is one of the most important factors that can alter the bioavailability of orally administered anticancer agents that are substrates of CYP3A [110]. A number of herbal supplements including *Echinacea*, kava, grape seed, and St John's wort (*Hypericum perforatum*) are also considered to be inducers of CYP [111] (Figure 1). Because of the increased use of herbal products by cancer patients, more consideration needs to be given to their combined use with anticancer agents [109, 112]. The administration of St. John's wort was shown to induce intestinal and hepatic expression of CYP3A [113] and be beneficial for the metabolism of irinotecan [114], a camptothecin derivative that can result in DNA damage on interaction with topoisomerase I. St. John's wort is hence used empirically in the treatment of metastatic carcinoma of the colon or rectum.

Recent studies based on epidemiological modeling have demonstrated interesting patterns suggesting that herbal treatment may improve prognosis in advanced colon cancer patients when used as an adjuvant therapy [115, 116]. The therapeutic mechanisms of traditional Chinese medicine in metastatic cancer have been discussed in terms of a hypothetical, dualistic antiproliferation and immune-stimulation model of tumor progression and regression [117]. Clinically, between 30% and 75% of patients with colon cancer are estimated to use CAM, but systematic or statistical evidence of survival efficacy is still limited. In one study with a 10-year followup of colon cancer patients (*n* = 193) who presented to a San Francisco Bay Area Center for Chinese medicine, authors compared the survival rate in patients choosing a short-term treatment regime lasting for the duration of their chemotherapy/radiotherapy period with those choosing a continuing long-term treatment. They also compared the survival of patients treated with Pan-Asian medicine plus vitamins (PAM+V) with that of concurrent external controls from the Kaiser Permanente Northern California and California Cancer Registries [118]. In this study, some modern methods, including Kaplan-Meier and traditional Cox regression, were used for analyses of causal inference, namely, propensity score and marginal structural models (MSMs), which have not been previously used in studies of cancer survival in response to treatment with Chinese herbal medicine. Results indicated that PAM+V combined with conventional therapy, as compared with conventional therapy alone, reduced the risk of patient death at stage I by 95%, stage II by 64%, stage III by 29%, and stage IV by 75%. No significant difference was observed between short-term *versus* long-term PAM+V administration [118]. This was apparently a sound clinical investigation and suggests that prospective trials combining PAM+V with conventional chemotherapy/radiotherapy may be clinically justifiable in future systematic studies.

Accumulating clinical studies show that some TCM preparations, including Pi-Sheng Decoction and Yi-Qi-Zhu-Yu Decoction, may be useful in reducing side effects and enhancing the drug effect of chemotherapy for colorectal cancer [119]. For preventing recurrence and metastasis, Jian-Pi-Xiao-Liu Decoction, Fu-Zheng Capsule, and Qu-Xie Capsule were used to decrease the recurrence and metastasis of colorectal cancer in a subsequent consolidation therapy after radical resection of patient's tumor. For improving the quality of life, Jian-Qi-Jie-Du Decoction, Jian-Pi-Yi-Qi Decoction, Fu-Pi-Yi-Wei Decoction, and Ai-Di injection were reported to enhance the antitumor “curative” effect of chemotherapy, reduce the side effects of chemotherapy, improve the immune function, and extend survival time in colorectal cancer patients. However, with the advancement of colorectal cancer treatment model, TCM theories and clinical studies on the typing of syndrome differentiation apparently are still lagging behind. In addition, current studies often have not addressed the issues on the anticancer properties or the observed beneficial health maintain/survival effects of treated TCMs. It is not only desirable, but also in fact necessary to further study the action model and the associated biochemical and physiological mechanisms of these anticancer mode herbs, as a milestone for future TCM research [119].

### 2.4. For Hepatocellular Carcinoma

The traditional Chinese medicine term or pathological classification of unresectable hepatocellular carcinoma (UHCC) is “liver stasis” [120]. Many clinical studies from China have indicated that TCM, such as Shentao Ruangan pills and hydroxycamptothecin, plus chemotherapy can significantly alleviate the symptoms, enhance therapy tolerance, stabilize tumor size and augment immunological function, reduce the incidence rate of adverse events, and prolong survival time of UHCC patients [121–123]. Although these studies may be criticized individually for lacking quality at the international level, together they do seem to suggest that the administration of TCM may warrant additional trials for patients with UHCC. Future clinical trials with TCM for UHCC need to have sufficient methodological quality and should be pursued in accordance with the Consolidated Standards of Reporting Trials (CONSORT) statement (see Section 3). In particular, rigorously designed, multicenter, large, randomized, double-blind, controlled trials are necessary [124].

### 2.5. For Other Cancers

Over the past two decades, a number of Chinese herbal medicines have been noted for their radiosensitization and radioprotection effects during radiotherapy of cancers, including bone cancer as well as head and neck tumors [125]. Cho and Chen [126] reported that a combination of TCM with radiotherapy not only enhanced therapeutic outcomes, but also improved the performance status of patients with nasopharyngeal cancer. Su and colleagues [127] consistently found that Guliu capsules (GLC) combined with Sr-89 conferred therapeutic effects in the treatment of metastatic bone tumors. They found that combined GLC and Sr-89 treatment was effective against metastatic bone tumor and improved patients' QOF enhancing ostalgia relief rate and decreasing hemotoxicity. In brain tumor treatment, Quan and colleagues [128] also reported that TCM, in combination with radio- or chemotherapy, had an effect on tumor growth inhibition, survival time, and QOL enhancement in brain tumor metastases. These findings further indicate the potential application of TCM in the therapy of different cancers (Figure 1).

### 2.6. Challenges for the Use of Herbal Medicine in Cancer Therapy

Although traditional herbal medicines, phytomedicines, medicinal foods, and complementary or alternative medicines have been increasingly used over the past decade in European and North American countries, they seem to have not generated interest or been accepted by mainstream medicine practitioners in western countries, especially in standard care for cancer patients. The key issue considered by many biomedical scientists has been the lack of evidence-based information/guidelines for routine and regulatory application of herbal medicines as “drugs” for use in public health. The sticking points hindering the use of phytomedicines can be attributed to six major issues: (1) lack of consistent and reliable sources of authentic medicinal plant materials, with respect to species verification and authentication, cultivation using good agricultural practice protocols, and standardized/normalized methods and technology for plant extraction/mixture preparation; (2) lack of definitions and routine preparation of the biochemical/biological ingredients and compositions of herbal medicines or the phytochemicals/phytocompounds derived from medicinal plants, with respect to identification of metabolite profiles, index compounds, and putative active compounds or metabolites; (3) general and specific safety considerations, including tolerable high dosage, minimal effective dosage, and specific usage; (4) proof of efficacy in treating or assisting specific cancer patients, including lack of results/data from preclinical animal studies, execution of bona fide, and double-blind, placebo-included, statistician-assisted clinical trial studies; (5) highly complex “personalized” prescriptions or formulations for the use of some traditional medicines (e.g., in TCM) that may be mystified by a “secret ingredient” in specific formulations; and (6) the criminal act of supplementation/ “spiking” highly potent western chemical drugs into herbal medicines in counterfeit activities. Without addressing all of the above issues, we cannot meet the challenges of modernizing herbal medicines. Although we have reviewed a spectrum of laboratory, preclinical and clinical studies on potential applications of herbal medicines for cancer patients' care in an inclusive fashion, a great many of these studies did not follow the stringent requirements, procedures, and protocol needed for developing western style drug or medicinal foods. Systematic and correlated efforts among researchers of our scientific communities are therefore urgently needed.

It is also important to note that the central tenet in recent western medicine is that a drug should be composed of well-known chemical components or a pure single compound that selectively interacts with known and specific molecular target(s) in our body system. However, the search for single molecules that can modify single or highly specific key factors in a disease process is now recognized as a difficult and sometimes inappropriate strategy, because a large volume of studies on genomics, proteomics, and metabolomics studies have shown that many clinically used commercial drugs (e.g., aspirin, doxorubicin, etc.) may in fact bind and work on multiple molecular targets. Multiple cell types, target molecules, and/or multiple signaling pathways are known to contribute to various diseases. Herbal extracts/mixtures prepared as traditional phytomedicines represent a combinational chemistry and “thus claimed” to encompass a vast and useful repertoire of chemical entities that can confer a complex and yet integrated effect on a spectrum of molecular and cellular components and functions, resulting in a profound and balanced medicinal activity. Unfortunately, according to the current FDA and NIH cancer clinical trial regulations in USA, such “claims” often directly conflict with the present guidelines or guidance. One major drawback in the integration of herbal medicines into mainstream western medicines is, therefore, the lack of defined molecular targets. With regard to this concern, recent research findings revealed from a spectrum of omics studies strongly suggest that a multifactorial mode of action and multitarget pharmaceutical activity may in fact already be the “norm” for a spectrum of currently used clinical drugs. As a result, there may be much less difference in terms of the complexity of molecular targets aimed by single compound drugs versus complex herbal medicine extracts than was originally assumed, as we previously demonstrated in a cancer cell line study [129]. We may then further project that the “multi-target” approach or activity believed for various herbal medicines may in fact be “reasonable and understandable” and therefore be positively considered and prepared in botanical drug development. Pooling data from individual trials by using a meta-analysis approach may be a useful strategy to interpret at the results of a group of inconclusive trials [130].

The uncertain or not well-defined composition of herbal products also raises questions about their safety, such as the evidence indicating that some herbal extracts may have harmful interactions with specific prescription drugs [131]. To address this issue, the establishment of optimized CMC (chemistry, manufacture, and control) conditions for each herbal preparation will need to be considered as important technology for confirming and standardizing the composition of specific medicinal herb components. Toward this aim, we believe that the pattern-oriented approach (fingerprint analysis) is a good strategy, because it can evaluate the integrative and holistic properties of test herbal medicines by comparing the similarities, differences, and correlation of the results from analyses of the whole production process, including manufacture, processing and storage of raw materials for preparation, intermediate products, finished products, and the distribution products [132, 133]. Yongyu and colleagues [134] have systematically reviewed fingerprint methods for analyses of herbal medicines. The fingerprint profiling of therapeutically used herbal medicines can be employed as a reference or index for quality control of phytochemical composition, and the results can be used in future clinical applications. Furthermore, the fingerprinting profiles can also be coordinated with and employed for therapeutic efficiency. This study approach was recently evaluated by an investigation of randomized controlled clinical trials (see Section 3).

In order to treat specific diseases, it is desirable that “modern drugs” can be generally applied to most patients with the same disease, although personalized medicine is becoming more popular. In traditional herbal medicines, mixtures of herbal extracts, comprising multiple phytocompounds, presumably regulating multiple targets for two or three medical indications are often used in a prescription. A major challenge for clinical use of such herbal remedies in cancer therapeutics is the evaluation of “true” active components and their targets for such multiple indications. Although modern chemical drugs and conventional herbal medicines may seem to be very different, they may, in fact, share some pharmacological foundations. As some herbal medicine classes have a common structural scaffold, this similarity may account for their potency in similar target groups [135]. It is believed that these structures and the activity/function information will become one of the most important indices for medicinal chemists to efficiently classify and seek specific pharmaceutical activities and for effectively optimizing herbal chemicals.

## 3. Evidence of the Effect of Herbal Medicines in Randomized Controlled Trials 

Randomized controlled trials (RCTs) (or randomized comparative trials) currently serve as the gold standard for most clinical trials and provide the best evidence of the efficacy of healthcare interventions [136]. Carefully planned and well-executed RCTs often obtain the best estimates of treatment effect and thus help guide clinical decision making; therefore, considerable effort is put into improving the design and reporting of RCTs [137, 138]. Linde et al. [139] commented that reporting quality may vary across different types of complementary therapies, with herbal medicine trials being apparently superior to homeopathy and acupuncture trials. Also, reporting quality differed among different individual botanical medicines and improved continuously for decades from the 1980s to the 2000s [140]. With these controversies, in June 2004 an international group of pharmacologists, methodologists, pharmacognosists, and trialists met for a consensus-making meeting, which then led to the development of recommendations for the reporting of herbal medicine trials in Toronto, Canada [141]. An elaboration of CONSORT statement was put forward that aimed to aid researchers to more accurately assess the internal/external validity and reproducibility of herbal medicine trials, to allow a more accurate assessment of safety and efficacy of herbal medicines [141, 142]. Among the 22 CONSORT checklist items, 9 of them were elaborated to enhance their relevance to the trials of herbal interventions, including the detailed recommendations for 1 item (item 4 (interventions)) and minor recommendations for 8 items (item 1 (title and abstract), item 2 (background), item 3 (participants), item 6 (outcomes), item 15 (baseline data), item 20 (interpretation), item 21 (generalizability), and item 22 (overall evidence)) [141]. Specifically, the detailed recommendation in item 4 addressed the concerns of the herbal medicine intervention, which is still need extensive elaboration. These recommendations have been developed to improve the reporting of RCTs.

Although TCM and other herbal medicines are being used worldwide, their efficacies have only been studied in a sporadic way, with very few properly randomized and controlled studies. Trials of note that have employed a high standard of clinical trial methodology include Mok and colleagues [143] and Chan and colleagues [144]. Mok and colleagues examined the possible role of Chinese herbal medicine in reducing chemotherapy-induced toxicity. They reported that traditional Chinese herbal medicine seemed to have a significant effect on the control of nausea in patients with early-stage breast or colon cancers, but these herbal medicines did not reduce the hematologic toxicity associated with chemotherapy. In addition, Chan et al. [144] conducted a randomized, placebo-controlled trial to evaluate the efficacy of test TCMs in improving QOL and reducing chemotoxicity and possible decrease in the side effects of systemic chemotherapy and the immune system status of patients undergoing a standard treatment for ovarian cancers. In this study, ovarian cancer patients were randomized to receive either the test TCM formulation or a placebo in addition to standard chemotherapy. The primary outcome was recorded by the global health status (GHS) score and assessed by the European Organization for Research and Treatment of Cancer questionnaire, and the secondary outcomes were examined using other QOL items, chemotoxicity levels defined according to the World Health Organization (WHO) criteria, and alterations in specific immune functions. The results suggest that TCM exerted effects in maintaining immune function (e.g., lymphocyte count and cytokine activities) rather than improving QOL. However, as these randomized trials failed to recruit sufficient study numbers, we may need to conclude that, in order to fully evaluate and demonstrate specific bioactivities and the merits of various TCM formulations or plant extracts in cancer patients, continued, systematic efforts in conducting scientifically sound studies with RCTs are required [144].

## 4. Other Anticancer Applications of Specific Herbal Medicines

Tumor microenvironments are now recognized to play a critical role in cancer growth, progression, and metastasis [145, 146]. Intensive interactions between tumor or cancerous cells and their stromal microenvironments that involve a spectrum of immune cell types have received considerable research attention over the past few years [146–148]. There has been particular interest in the strong link between various immune activities at or surrounding tumor tissues and the progression of tumor growth. Enhancement of tumor surveillance by the host immune system has also been considered to be a key strategy to facilitate anticancer effect. In this section, we address the specific effects of herbal medicines on the enhancement of host immunity and review their molecular targets in anticancer activities.

### 4.1. Herbal Medicines as Adjuvant for Dendritic Cell-(DC-)Based Vaccines

By definition, the function of an adjuvant used with a vaccine is to aid or promote antigen delivery and presentation. An adjuvant can also assist in the induction of cytokines and stimulation/activation of antigen-presenting cells in the tumor or tissue microenvironment [149]. Specific herbal medicines such as *Ganoderma lucidum* or Dioscorea tuber have been reported to confer immunomodulatory activities [150, 151]. Bioactive polysaccharides from *Ganoderma lucidum* (Reishi) were investigated for their immunostimulatory and anticancer properties [152]. A specific polysaccharide fraction from Reishi stimulated immune cell activation including dendritic cell maturation and cytokine expression and displayed potent adjuvant activity in mice [153, 154]. Polysaccharides from *Dioscorea batatas* were found to induce TNF-*α* secretion via Toll-like receptor 4-mediated protein kinase signaling pathways [155]. A number of phytochemicals have also been demonstrated to effectively enhance the anti-tumor potency of gene-based cancer vaccines. For example, shikonin enhanced the anti-tumor potency of a cancer vaccine via the induction of RANTES expression at the skin immunization tissue site [156]. And a phytocompound mixture extracted from the butanol fraction of a stem and leaf extract of *Echinacea Purpurea* conferred immunomodulatory effects suggesting that it can effectively modulate DC mobility and related cellular physiology *in vivo* in the mouse immune system [157]. These studies suggest the potential application of herbal medicines in a cell-based vaccine system.

### 4.2. Induction of Immunogenic Cell Death by Herbal Medicines

Immunogenic cell death mediated by damage-associated molecular pattern (DAMP) signals was found to trigger an immunogenic response including maturation and antigen uptake of dendritic cells [158]. Recently, Chen and colleagues [159] demonstrated that shikonin can induce immunogenic cell death in treated tumor cells. Shikonin-treated, tumor cell lysate-loaded, mature DCs were shown to exhibit strong anticancer activities against test mouse melanoma, including the induction of cytotoxic activities of splenocytes against target tumor cells, inhibition of tumor growth, and improvement in mouse survival. The use of shikonin-treated tumor cells from patients to pulse their own DCs in culture should be evaluated in future clinical studies as a new approach for developing DC-based anticancer vaccines.

## 5. Conclusion and Future Development

For centuries if not millennia, various plants (many systematized in traditional Chinese medicine) have been used as medicines and disease therapeutics in most human cultures. As exemplified in this review, over the last two decades renewed public interest and research efforts from scientific and medical communities worldwide have generated a large volume of information including clinical studies and trials on the pharmacological effects, usage, and the development into future medicines of herbs and derivative medicinal phytochemicals as anti-tumor and chemoprevention agents. Although considerable effort has been put into the verification and upgrade of many traditional remedies or multiple-herb formulations, systematic, standardized research and the use of FDA regulatory protocols and defined clinical trials are still quite limited and need to be actively pursued. At the same time, it is necessary for scientists, clinicians, and regulatory agencies to actively consider how to create novel, improved, or modified clinical surveys, studies, and trial mechanisms that employ the stringent trial standards of the 21st century but also incorporate, at the international level, the wealth of old empirical but incomplete data from various records and documents accumulated by traditional medicine practices worldwide, to expedite the discovery and development of new phytomedicines and botanical drugs.

While continuous and systematic effort is needed, a number of notable “breakthroughs” have occurred in the field of medicinal plant research and botanical drugs in the last few years. In April 2008, the FDA approved the very first botanical drug, Veregen, a partially purified fraction of the water extract of green tea leaves from *Camellia sinensis*, for topical treatment of external genital and perianal warts [160]. Very recently (January 2013), the FDA approved, for the first time, an oral botanical drug, Crofelemer (a purified oligomeric proanthocyanidin from the latex of the South American *Croton lechleri* tree), for treatment of diarrhea in HIV/AIDS patients. Although these two pioneer FDA-approved botanical drugs are not therapies for cancer, they certainly pave way for such future developments. One possible example is the ongoing (2013) FDA clinical trial on “PHY906.” This four-herbal-plant-composed TCM formulation has been shown to confer with good evidence [74]. It is our hope that the phase III clinical trial of this formula will lead the way in the development of CAM for cancer patients. With the various other new clinical trials ongoing, CAM may start playing critical roles in future health care of aging populations.

## Figures and Tables

**Table 1:** Clinical use of herbal medicines exhibiting anticancer activities.

Herbal medicine	Suppressive effects on carcinogenesis and cancer metastasis	References
For breast cancer		
Vitamin A (fenretinide)	200 mg/day significantly reduces the recurrence of local breast cancer in premenopausal women	[4]
Vitamin E	Leads to malabsorption or maldigestion in cancer patients; balanced and healthy diet	[5, 6]
Isoflavone	To reduce risk of breast cancer	[10]
Isoflavones genistein and daidzein	To confer weak estrogenic effects	[11]
Alkaloids	Inhibition of cancer cell growth	[13, 14]
Coumarins	Inhibition of cancer cell growth	[15, 16]
Flavonoids and polyphenols	Antiproliferation	[17, 18]
Terpenoids	MCF-7 cell apoptosis	[19]
Quinone	To induce G2-M arrest and autophagy by inhibiting the AKT/mammalian target of rapamycin pathway in breast cancer cells	[20]
Artemisunate	Decrease the proliferation of human breast cancer cells from expressing a high ER*α* : ER*β* ratio	[21]

For prostate cancer		
Vitamins A-D and retinoid	Maintain homeostasis and prevent various metabolic disorders	[23]
Vitamin E	Reduce the risk of lethal or advanced prostate cancer relative to nonusers	[30]
Epigallocatechin-3-gallate (EGCG)	Arrest LNCaP and DU145 prostate cancer cells at the G0-G1 phase of the cell cycle	[34]
	Inhibit metalloproteinase *in vitro *	[35]
Soy isoflavones	Inhibit 5*α*-reductase activity	[37]
	Chemopreventive activities	[22]
*Scutellaria baicalensis* (baicalin)	Inhibit enzymatic synthesis of eicosanoids	[38]
Baicalein	Impair the proliferation of androgen-independent PC-3 and DU145 prostate cancer cells in culture	[39]
	Induces cell-cycle arrest at the G0-G1 phase	[39]
	Induces apoptosis of prostate cancer cells at concentrations achievable in humans	[40]
	Suppresses the expression of specific androgen receptor in prostate cancer	[40]
Lycopenes	Decreases prostate cancer risk	[41]
	Diminishes oxidative damage in lymphocytes	[42]
	Significantly decreases levels of PSA and less oxidative damage	[42]
PC-SPES	Decreases serum testosterone concentrations (*P* < 0.05); decreases serum concentrations of prostate-specific antigen	[43]
	Antitumor efficacy against cancer cell lines	[44]
*Wedelia chinensis* (Asteraceae)	Inhibits the androgen receptor (AR) signaling pathway	[47]

For lung cancer		
*Platycodon grandiflorum* (Campanulaceae)	Anticancer effect in lung cancer patients	[49–51]
*Morus alba* (Moraceae)	Anticancer effect in lung cancer patients	
*Prunus armeniaca* (Rosaceae)	Anticancer effect in lung cancer patients	
*Rhus verniciflua* (Anacardiaceae)	Anticancer effect in lung cancer patients	
*Perilla frutescens *(Labiatae)	Anticancer effect in lung cancer patients	
*Stemona japonica *(Stemonaceae)	Anticancer effect in lung cancer patients	
*Tussilago farfara *(Compositae)	Anticancer effect in lung cancer patients	
*Draba nemorosa* (Brassicaceae)	Anticancer effect in lung cancer patients	

For liver fibrosis and cancer		
*Inchin-ko-to* (TJ-135)	Preventive effect on liver fibrosis	[53]
*Yi Guan Jian* (YGJ)		[53]
*Yi Guan Jian* (YGJ)		[54]
*Fufang-Liu-Yue-Qing *		[55]
*Danggui Buxue Tang *(DBT)		[56]
Salvianolic acid B		
Curcumin	Suppressive effect on hepatic fibrogenesis and carcinogenesis	[57]
Oxymatrine		
Compound 861	Suppressive effect on hepatic fibrogenesis	[58, 59]
*Sho saiko-to* (TJ-9)	Reduces/limits the progression of hepatocellular carcinoma	[60]

For pancreatic cancer		
GDC-0449, IPI-926, XL-139 and PF-04449913	SMO antagonists; deregulation of sonic hedgehog homology (SHH)	[61]
Cyclopamine	Inhibit SHH signaling by directly binding to the 7-helix bundle of the SMO protein; arrest the growth of pancreatic tumors	[62]
	Weakens the recruitment of BMPCs into cancer cells and reduces the formation of tumor vasculature	[63]
	The cancerous vascular system becomes unstable after treatment with cyclopamine due to the expression of angiopoietin-1	[63]
